# Advancement of fluorescent aminopeptidase probes for rapid cancer detection–current uses and neurosurgical applications

**DOI:** 10.3389/fsurg.2024.1298709

**Published:** 2024-03-07

**Authors:** Takenori Shimizu, Shota Tanaka, Yosuke Kitagawa, Yusuke Sakaguchi, Mako Kamiya, Shunsaku Takayanagi, Hirokazu Takami, Yasuteru Urano, Nobuhito Saito

**Affiliations:** ^1^ Department of Neurosurgery, Graduate School of Medicine, The University of Tokyo, Tokyo, Japan; ^2^ Department of Neurosurgery, Massachusetts General Hospital, Harvard Medical School, Boston, MA, United States; ^3^ Department of Life Science and Technology, Tokyo Institute of Technology, Tokyo, Japan; ^4^ Laboratory of Chemical Biology and Molecular Imaging, Graduate School of Medicine, The University of Tokyo, Tokyo, Japan; ^5^ Laboratory of Chemistry and Biology, Graduate School of Pharmaceutical Sciences, The University of Tokyo, Tokyo, Japan

**Keywords:** hydroxymethyl rhodamine green, γ-glutamyl-HMRG, glutamylprolyl-HMRG, glioblastoma, glioma, 5-aminolevulinic acid

## Abstract

Surgical resection is considered for most brain tumors to obtain tissue diagnosis and to eradicate or debulk the tumor. Glioma, the most common primary malignant brain tumor, generally has a poor prognosis despite the multidisciplinary treatments with radical resection and chemoradiotherapy. Surgical resection of glioma is often complicated by the obscure border between the tumor and the adjacent brain tissues and by the tumor's infiltration into the eloquent brain. 5-aminolevulinic acid is frequently used for tumor visualization, as it exhibits high fluorescence in high-grade glioma. Here, we provide an overview of the fluorescent probes currently used for brain tumors, as well as those under development for other cancers, including HMRG-based probes, 2MeSiR-based probes, and other aminopeptidase probes. We describe our recently developed HMRG-based probes in brain tumors, such as PR-HMRG, combined with the existing diagnosis approach. These probes are remarkably effective for cancer cell recognition. Thus, they can be potentially integrated into surgical treatment for intraoperative detection of cancers.

## 1 Introduction

Surgical resection is the primary treatment for brain tumors, complemented by radiotherapy or chemotherapy for malignant types (1–3). Total resection is often challenging, especially for gliomas, due to their infiltrative nature and difficulty in distinguishing from surrounding tissues (4–7). Fluorescence imaging, rapidly adopted in neurosurgery, addresses these challenges. It offers low-cost, high-resolution visualization of tumors, clearly differentiating them from adjacent brain tissue and aiding in the identification of ill-defined boundaries. This method is particularly crucial in reducing residual tumors and the associated risk of regrowth or relapse (8–11).

Panel diagnostics using next-generation sequencing have advanced the identification of oncogenes in solid cancers, such as lung and breast cancers, paving the way for precision medicine (12, 13). While these omics analyses provide comprehensive insights, they lack spatiotemporal data after cell homogenization. In contrast, fluorescence imaging in surgery offers a non-invasive, real-time, and high-resolution method for observing and quantitatively analyzing biomolecules within tissues (8, 9).

Fluorescent probes can be broadly categorized based on their features (14). The “always on” probes continuously exhibit fluorescence, whereas “activatable” probes only become fluorescent upon interaction with a specific target. Today, “always on” probes such as indocyanine green (15) and fluorescein sodium are used in the neurosurgical field (9, 16). However, these probes are not always accumulating in tumor tissues and have tendency to emit high background from the effect of “always on” (9). On the other hand, 5-aminolevulinic acid (5-ALA) serves as an “activatable” probe, distinguishing the tumor from the non-tumor tissues based on metabolic activity variations (8).

This review highlights advancements in fluorescent aminopeptidase probes, especially Hydroxymethyl Rhodamine Green (HMRG), 2-Methyl silicon rhodamine (2MeSiR), and 2-O-Methyl silicon rhodamine (2OMeSiR). These probes are expected to enhance surgical precision and rapid cancer detection. Their interaction with tumor enzymes allows accurate differentiation between tumor and surrounding tissues. We will explore their benefits and applications in fluorescence-guided surgery.

## 2 HMRG-based aminopeptidase probes for various cancers

**Figure 1:**
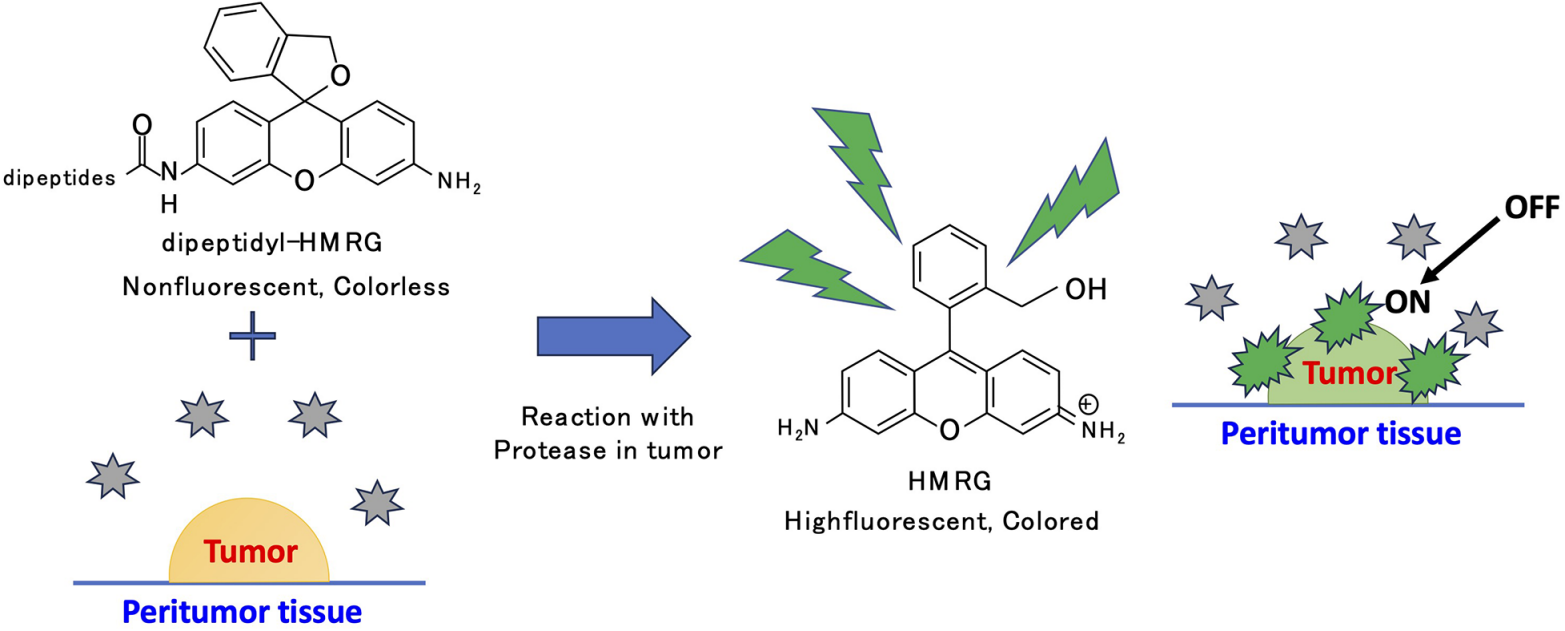
Chemical structure and reaction principle of HMRG probe. When dipeptidyl-HMRG reacts with the enzyme in tumor, it becomes HMRG and gets fluorescent. HMRG distinguishes tumor from the non-tumor tissue as an “activatable” probe.

Miura et al. reported a rational design principle, Photoinduced Electron Transfer, for modulating the fluorescence properties of probes using fluorescein (17). Building upon this theory, Urano et al. synthesized probes with 2,6-dicarboxyethyl-1,3,5,7-tetramethyl boron-dipyrromethene (BODIPY) as a fluorescent scaffold, enabling the detection of acidic pH changes *in vitro* with HER2-positive cells. Significantly, these probes possessed the added advantage of reversibility, making them suitable for *in vivo* applications in lung cancer detection (18). To address the slower reaction rate of endo-peptidase detectable probes that hydrolyze non-terminal peptides, we employed intramolecular spirocyclization (19). This concept facilitated rapid responsiveness and precise molecular design, targeting the amino-terminal or carboxyl-terminal ends (19–21). Furthermore, Kuriki et al. established HMRG-based probe libraries that potentially targeted different enzymes achieved by substituting the acetyl group of Ac-HMRG with various amino acids (22) (Figure 1).

### 2.1 γ-Glutamyl (gGlu)-HMRG probe

Urano et al. applied gGlu-HMRG to human ovarian cancer cell lines (SHIN-3) and normal human umbilical vein endothelial cells, observing high fluorescence intensity and γ-glutamyltransferase (GGT) activity exclusively in SHIN-3 cells (23). gGlu-HMRG, reacting with GGT expressed on tumor cells, produces a highly fluorescent reaction product (24, 25). In experiments with mice bearing SHIN-3 tumors, intraperitoneal injection of gGlu-HMRG led to distinct high fluorescence in tumor areas, visible to the naked eye, while normal mice without tumors showed no such fluorescence and had low GGT background activity. This fluorescence was also confirmed *in vitro* with many human ovarian cancer cell lines tested. Spraying gGlu-HMRG on the peritoneal surface of mice injected with six of these cell types resulted in strong fluorescence in four types. In further studies, SHIN-3 cells transfected with red fluorescent protein (RFP) and injected into mice revealed complete overlapping fluorescence with gGlu-HMRG 10 min post-injection. Notably, the detection of SHIN3-RFP cells using gGlu-HMRG showed 100% sensitivity and specificity (23).

### 2.2 Glutamyl-prolyl (EP)-HMRG probe

Dipeptidyl peptidase IV (DPP-IV), a ubiquitous enzyme found in the kidney, liver, intestine, and other organs, is implicated in various cancers including prostate adenocarcinoma, thyroid cancer, and esophageal cancer (26–29). It can cleave EP-HMRG, resulting in fluorescence emission (30).

Onoyama et al. developed peptidase probes for esophageal squamous cell cancer (30). Screening was performed using a series of HMRG-based aminopeptidase-activatable fluorescence probes such as γ-glutamyltranspeptidase, DPP-IV, fibroblast activation protein (AcGP-HMRG), cathepsin H (Arg-HMRG), against fresh biopsy samples. They discovered that glycine-prolyl-HMRG (GP-HMRG), targeting DPP-IV, exhibited a rapid, substantial, and specific increase in fluorescence in tumor cells. After assessing DPP-IV expression levels and enzymatic activity, they synthesized HMRG probes with various N-terminal amino acids, such as Glutamic acid, Lysine, Tyrosine, Leucine, and Proline, to determine their affinity. The EP-HMRG probe, showing the highest affinity and lowest Michaelis constant (Km) for DPP-IV, was selected. Validation of EP-HMRG involved measuring the increase in fluorescence intensity in tumor and normal samples over time, revealing a significant increase in tumor samples.

### 2.3 Proline-arginine (PR)-HMRG probe

**Figure 2:**
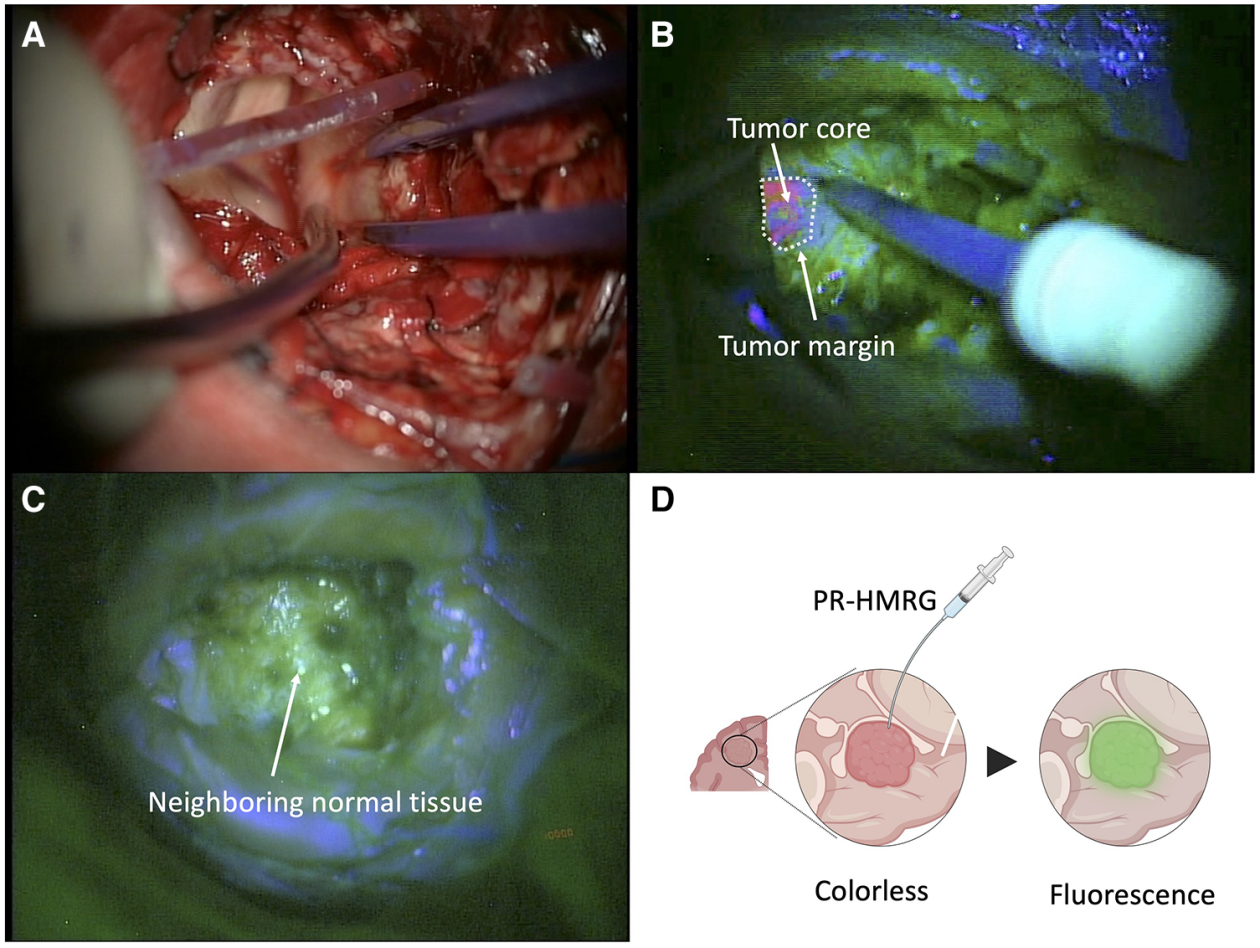
Fluorescence-guided resection using 5-ALA and PR-HMRG in glioma surgery. (**A**) Corticotomy site visualized under white light. (**B**) Visualization of the tumor core and margins using 5-ALA-induced fluorescence at an emission wavelength of approximately 630 nm. (**C**) Post-resection view showing no residual fluorescence, suggesting complete removal of the tumor. (**D**) Schematic representation of PR-HMRG probe activation, depicting the transition from a colorless state to fluorescence upon enzymic reaction with the tumor.

Recently, we published our research on the development of fluorescent probes designed for glioblastoma, relying on enzymatic activity (31). We initially screened 320 fluorescent probes using homogenized tumor lysates from patients, selecting the top 10% of promising probes based on their ability to differentiate between glioblastoma and surrounded tissues. We further narrowed down these candidates in a secondary screening with fresh surgical specimens, identifying the top three probes that demonstrated the highest differential fluorescence intensities for glioblastoma detection. These results were comprehensively analyzed, and a tertiary screening involved computational, mathematical, and pathological analysis. The proline-arginine-HMRG (PR-HMRG) probe showed the highest reactivity with 79.4% accuracy for detecting glioblastoma. We also attempted to identify the enzyme-cleaving PR-HMRG using Diced Electrophoresis Gel (DEG) assay, followed by Liquid Chromatography/Tandem Mass Spectrometry (LC/MS) (32). Through LC/MS, we identified four potential enzymes, with calpain 1 (*CAPN1*) as the responsible one, confirmed by enzyme inhibition experiments and *CAPN1* RNA expression analysis. In U87 glioblastoma cells, *CAPN1* knockdown reduced PR-HMRG fluorescence (31). In a U87 orthotopic xenograft model, PR-HMRG displayed higher fluorescence in tumor areas, consistent with *CAPN1* expression. Human surgical specimens also showed elevated *CAPN1* expression by both immunohistochemistry and western blotting, indicating the potential of this probe for glioblastoma detection during the surgery in the future (31). PR-HMRG probe showed early fluorescence onset within 5 min of application (31) (Figure 2).

### 2.4 Other HMRG-based probes

Kuriki et al. found promising probes lysine-histidine-HMRG (KH-HMRG) for gastric cancer (negative staining) and lysine-lysine-HMRG (KK-HMRG) for lung cancer from among 380 types of HMRG-based fluorescent probes, which detect tumor tissues with high expression of puromycin-sensitive aminopeptidase (PSA) and aminopeptidase N (APN), respectively (22).

Takahashi et al. selected the promising fluorescent probe GP-HMRG for pancreatic cancer from our probe library (33). Dipeptidyl peptidase, or DPP-IV-like enzyme, was identified as the target enzyme.

Ac-lysine-glutamine-leucine-arginine-HMRG (Ac-KQLR-HMRG) is a fluorescent probe for visualizing prostate cancer. Yogo et al. synthesized Ac-KQLR-HMRG, which is activated by hepsin and matriptase. This probe showed specific fluorescence of various prostate cancer cell lines *in vitro* (34).

Yamamoto et al. synthesized an avidin-conjugated fluorescent probe, the Avidin-Leu-HMRG (35). Avidin is a protein which has a high affinity for lectin on cancer cells. In a mouse model of peritoneal ovarian metastasis, this probe demonstrated high fluorescence intensity at tumor locations, attributable to the fluorescence activity of lysosomal leucine aminopeptidase.

HMRG-based fluorescent probes may be useful for various diseases other than cancers. Yamashita et al. evaluated the fluorescence intensity in pancreatic juice and intestinal juice discharged after the pancreatic ventricle or central pancreatectomy using glutamyl-phenylalanine-HMRG (gPhe-HMRG) (36). They showed that it is possible to measure protease (chymotrypsin) activity in drained pancreatic fluid samples.

## 3 2MeSiR and 2OMeSiR probes

Challenges with green HMRG probes include interference from tissue autofluorescence and attenuation related to blood absorption (37). To circumvent these limitations, researchers have identified alternative scaffolds that emit at longer wavelengths. Kushida et al. demonstrated that 2MeSiR600, a red fluorescent scaffold, could be used to design activatable probes targeting proteases, although it exhibited high background fluorescence due to its relatively high fluorescence quantum yield (38). Addressing this, Ogasawara et al. modified 2MeSiR600 to reduce background signals and synthesized 2OMeSiR600 probes for aminopeptidase activity detection, controlled by photoinduced electron transfer (39).

### 3.1 Proline-methionine-2MeSiR (PM-2MeSiR)

Takahashi et al. developed a fluorescence imaging technique to identify the extrahepatic biliary tree (eCCA) using an enzyme-activated probe for diagnostic imaging of liver cancer (40). They selected the most specific probe for eCCA from 384 HMRG-based fluorescent probes, 400 2MeSiR-based probes, and 16 types of Hydroxymethyl N-(2,2,2-trifluoroethyl) rhodol (HMRef) -based fluorescence probes for glycosidases that they had synthesized (41). PM-2MeSiR emerged as the most specific fluorophore for eCCA, targeting a puromycin-sensitive aminopeptidase (40).

### 3.2 Glutamine-alanine-2MeSiR (QA-2MeSiR) and glutamine-alanine-2OMeSiR (QA-2OMeSiR)

QA-2MeSiR and QA-2OMeSiR are probes developed for detecting tumors in lung cancer. Kawashima et al. screened these probes, selecting those with the highest fluorescence intensity for lung cancer (42). They found QA-2OMeSiR to have a lower background than QA-2MeSiR, targeting enzymes like DPP-IV and PSA (42).

Table 1 shows a summary of the HMRG-based and 2MeSiR-based fluorescent probes.

**Table 1:** Details of HMRG, 2MeSiR and 2OMeSiR probes.

Fluorescent scafold	Abbreviation	Disease and event	Target enzyme
HMRG	gGlu-HMRG	Breast cancer, renal cortex, neck squamous cell carcinoma, oral cancer, lung cancer, hepatic cancer, colon cancer, prostate cancer, ovalian cancer, thymic carcinoma, diabetic Kidney disease and glomerular diseases	γ-Glutamyl transpeptidase
	EP-HMRG	Esophageal cancer	Dipeptidyl peptidase IV (DPP-IV)
	gPhe-HMRG	Postoperative pancreatic fistula	Chymotrypsinogen
	PR-HMRG	Glioblastoma	Calpain-1
	Ac–KQLR–HMRG	Prostate cancer	Hepsin and matriptase
	Avidin-Leu-HMRG	Prostate cancer	Lysosomal leucineaminopeptidase
	GP-HMRG	Pancreatic cancer	Dipeptidyl-peptidase IV
	KH-HMRG	Gastric cancer	Puromycin-sensitive aminopeptidase (PSA)
	KK-HMRG	Lung cancer	Aminopeptidase N (APN)
2MeSiR	QA-2MeSiR	Lung cancer	Dipeptidyl peptidase IV (DPP-IV)
	PM-2MeSiR	Extrahepatic biliary tree (eCCA)	Puromycin-sensitive aminopeptidase
2OMeSiR	EP-2OMeSiR	Esophageal cancer	Dipeptidyl peptidase IV (DPP-IV)
	QA-2OMeSiR	Lung cancer	Dipeptidyl peptidase IV (DPP-IV)

## 4 Other fluorescent aminopeptidase probes

Leucine aminopeptidase (LAP) is an enzyme that cleaves a type of amino acid from the end of a peptide. It has been confirmed that LAP's blood concentration increases due to bile stagnation, and LAP is present in various cancer cells (43). Gong Q et al. developed the fluorescent probe of the incorporating L-leucine into the skeleton of cresyl violet as a recognition moiety using confocal fluorescence imaging (44). They analyzed changes in LAP concentration using human liver cancer-derived HepG2 and human lung cancer-derived A549 cells under cisplatin treatment. A higher concentration increase of LAP was found in HepG2 cells. Inhibitor experiments of LAP expression with siRNA further reduced cell viability. This result indicated that LAP was highly resistant to cisplatin. LAP is known to be involved in detoxifying cisplatin in hepatoma cells and contributes to inherent drug resistance (44). He X et al. developed a specific and sensitive near-infrared fluorescent probe (HCAC) for *in vivo* imaging of LAP activity in liver disease models. HCAC showed acetaminophen-induced liver injury and upregulation of LAP in tumor mouse models (45).

Pyroglutamate aminopeptidase-1 (PGP-1) enzyme plays an important role in inflammation involving immune cells, blood vessels, and molecular mediators (46). Cao et al. designed a red-emitting ratiometric fluorescence sensor (DP-1) that specifically detects PGP-1. They showed that PGP-1 expression was associated with inflammation using human liver cancer-derived HepG2 and mouse macrophage-like cell line RAW264 cells by imaging of the DP-1. Furthermore, imaging of mouse tumor models has shown that PGP-1 is closely associated with some inflammation and tumor disease (46).

Prolyl aminopeptidases (PAP) are often present in infectious disease bacteria, which is a potential biomarker and therapeutic target for pathogen infection (47). Liu X. et al. developed a near-infrared fluorescent “turn-on” probe (NIR-PAP) for detecting and imaging the activity of PAP *in vivo*. They indicated that this probe exhibited high specificity and reactivity to PAP under physiological pH and temperature conditions *in vitro* (47).

APN is expressed in ovarian carcinoma cells and is an important biomarker for cancers such as osteosarcoma and hematopoietic tumors (48–50). NIR fluorescent probes have been developed for detecting APN activity. He X et al. have developed an NIP fluorescent probe detecting APN (51). Using confocal microscopy, they showed that hepatoma cells had higher APN content than normal cells. Additionally, APNs were imaged in cells and mice *in vivo*. CD3/aminopeptidase N is an ectoenzyme with multiple functions, including tumor growth, migration, angiogenesis, and metastasis. LiH et al. have developed the first two-photon NIR fluorescent probe for *in vitro* and *in vivo* tracking of APN (52). Hydrolysis of the amino group of the N-terminal alanyl moiety restored the intramolecular charge transfer effect, resulting in strong fluorescence. In addition, the probe DCM-APN distinguished normal cells (LD2 cells) from cancer cells (human liver cancer-derived HepG-2 and malignant melanoma B16/BL6 cells).

## 5 Discussion

The standard treatment for most brain tumors is surgical resection under a microscope, often accompanied by adjuvant radiotherapy and/or chemotherapy for malignant types (1–3). Maximal resection is attempted for prolonged tumor control and improved patient survival in most cases, except for certain tumors like malignant lymphoma and germinoma, which are sensitive to either radiotherapy or chemotherapy (53, 54). The utilization of fluorescent probes in surgical procedures offers a significant advantage by enabling surgeons to accurately differentiate tumor from normal or surrounding tissues in real-time (55–57). This enhanced visualization, provided by the fluorescence of these probes, leads to increased resection rates—a critical factor in surgical success. Importantly, achieving a higher extent of resection, especially Gross Total Resection (GTR), has been independently associated with improved progression-free survival (PFS) and overall survival (OS) in patients with high-grade and supratentorial low-grade gliomas. Therefore, by facilitating more precise and extensive tumor resections, fluorescent probes have the potential to further improve PFS and OS outcomes in brain tumor patients.

The development of aminopeptidase probes, particularly HMRG-based and 2MeSiR-based probes, presents promising advancements in cancer detection and monitoring as biomarkers. These probes offer unique advantages, such as rapid activation and reduced background signals. The HMRG probes react quickly, yielding results within minutes, a benefit already confirmed in esophageal cancer and brain tumor studies (30, 31). In their studies, probes that target enzymes like g-glu, DPP4, CAPN1, LAP, PGP-1, and APN hold significant promise for detecting a variety of diseases, encompassing both cancer and infections (58, 59). Present, three fluorescent agents that have been studied and utilized widely in human neurosurgical fields are fluorescein sodium, ICG, and 5-ALA (8, 9).

ICG is a water-soluble molecule that is excited at a wavelength of approximately 780 nm and emits fluorescent within the 700–850 nm range, making it detectable only with a filtered scope. ICG is observed a few seconds after 0.2–0.5 mg/kg IV administration, reaching its peak around 10 min (15, 60, 61). ICG is widely used to confirm blood flow and patency in vascular surgeries for aneurysms, AVMs, and anastomoses. It can also be beneficial in assessing the circulatory status when tumors compress or infiltrate cerebral circulation (62, 63). The Second Window ICG technique uses tumors' vascular permeability and poor clearance. Delivering substantial quantities of ICG allows neurosurgeons to locate tumors during surgery. However, it takes 19–30 h to visualize and does not accumulate in a tumor-specific manner (64, 65).

Fluorescein demonstrates fluorescence, peaking at around 530 nm when excited at approximately 480 nm with high detection rate for glioma in multicentric prospective phase II trial (66). For lower concentrations, observation through a 560 nm filter is typically required to detect its fluorescence (67). Notably, at higher doses, specifically 20 mg/kg, fluorescein's fluorescence becomes visible to the naked eye (68). The technique of confocal endoscopy and endomicroscopy, which has been employed with fluorescein, is notable for its application in brain tumor imaging (69, 70). ICG and fluorescein does not selectively activate fluorescence in malignant glioma cells. Instead, it tends to concentrate in areas where the blood-brain barrier is disrupted, a common characteristic of tumor sites (67, 71).

5-ALA can be used to visually distinguish tumor tissues from normal ones (72, 73). 5-ALA transforms into protoporphyrin IX (PpIX), which is a photosensitizer and precursor in heme synthesis. PpIX excites and emits at 405 nm (violet) and 633 nm, enabling broad-spectrum activation (74). PpIX accumulation results from increased 5-ALA levels, elevated 5-ALA synthase activity, or malfunctioning ferrochelatase (FECH) enzyme, facilitating its conversion into heme (75). Glioblastoma exhibits reduced FECH expression compared to normal brain tissue, contributing to PpIX accumulation (76). Instances of ventricular wall fluorescence, indicating false positives, are observed even in cases where magnetic resonance imaging (MRI) or macroscopic observation show no evidence of tumor involvement (77). Stummer et al. noted that 5-ALA was effective in increasing tumor resection rates to 65% and enhancing six-month progression-free survival to 41%, as opposed to lower rates without it. However, its fluorescence is stronger in high-grade gliomas but weaker in low-grade ones. The compound becomes fluorescent six hours after intake but loses potency over time as it is metabolized. Other disadvantages include the potential for false positives in cases of radio-necrosis or inflammation, and false negatives in low-density areas (78–82).

Recent advancements have led to the development of both flexible and rigid endoscopic systems that utilize 5-ALA fluorescence, thereby enhancing surgeons' capabilities in the diagnosis and resection of brain tumors. The flexible endoscope system is particularly adept at observing 5-ALA fluorescence, aiding in the accurate identification of tumor margins (83). Conversely, the rigid endoscope system, which has been commercially available and widely reported, demonstrates effectiveness in 5-ALA fluorescence-guided surgery, significantly contributing to surgical outcomes (84, 85). However, despite these significant advancements, the diagnostic utility of these endoscopic systems as adjuncts to microsurgery remains somewhat limited. The integration of confocal endomicroscopy with 5-ALA is proposed as a promising approach to overcome these limitations. This integration potentially allows for a more detailed and nuanced observation of brain tumors at the microstructural level, which could be particularly beneficial in cases of suspected low-grade gliomas (86, 87).

Economically, PR-HMRG, as a fluorescent-guided surgery, may provide a cost-effective alternative compared to the acquisition of other supportive equipment such as navigation systems, intraoperative MRI, or intraoperative ultrasound sonography (88, 89). This makes the initial cost relatively low, especially when integrated into existing systems designed for 5-ALA, leading to avoidance of the substantial initial investments associated with other advanced diagnostic imaging methods (88, 89). These microscopes are already fitted with the necessary light source and fluorescence display monitors. Utilizing the existing setup with a filter exchange avoids the significant costs associated with major equipment modifications. Integrating HMRG and 2MeSiR probes into neurosurgical microscopes equipped with 5-ALA systems involves switching the microscope's internal filters to match the specific excitation and emission profiles of these probes. HMRG requires blue light excitation at 488 nm for its green fluorescence emission at 524 nm, while 2MeSiR needs an excitation filter at 593 nm to enable its 613 nm red fluorescence emission (31, 39).

Recent advancements in aminopeptidase probes, particularly those based on HMRG and 2MeSiR, are showing significant promise in improving cancer detection and monitoring as disease biomarkers (90, 91). These probes offer distinctive advantages, such as rapid activation and reduced background signals. Probes targeting enzymes like GGT, DPP-IV, CAPN1, LAP, and APN demonstrate potential in detecting various cancers and infections. Ongoing research aimed at enhancing their accuracy and minimizing false results is crucial. Systematic reviews and meta-analyses will likely play a key role in evaluating these newer probes as they transition from preclinical to clinical applications. In summary, fluorescent aminopeptidase probes represent a promising advancement in tumor visualization and image-guided surgery.
